# Unveiling the Hidden Culprit: A Case Report on Tachy-Bradyarrhythmias Presenting as Seizure Disorders

**DOI:** 10.2174/011573403X344795250304084151

**Published:** 2025-03-20

**Authors:** Kirtivardhan Vashistha, Akshat Banga, Michael Nestasie, Sarumathi Thangavel, Mian Tanveer Ud Din, George Shaw

**Affiliations:** 1 Department of Cardiovascular Medicine, Mount Sinai Morningside - West, Mount Sinai Health System, New York, NY, 10025, USA;; 2 Department of Internal Medicine, Mount Auburn Hospital, Harvard Medical School, Cambridge, MA, 02138, USA;; 3 Department of Cardiovascular Medicine, Cardiovascular Institute, Allegheny Health Network, Pittsburgh, PA, 15212, USA;; 4 Department of Internal Medicine, Allegheny Health Network, Pittsburgh, PA, 15212, USA

**Keywords:** Arrhythmia, cardiogenic syncope, seizures, tachy-bradyarrhythmia, syncope, atrial fibrillation, ventricular fibrillation

## Abstract

**Background/Introduction:**

The misdiagnosis of seizure disorders in patients with cardiogenic syncope and tachy-bradyarrhythmias is a significant diagnostic challenge as the differentials for altered mental status and syncope are broad and can mimic other clinical conditions. This case report presents a unique case of an elderly male with life-threatening ventricular arrhythmia, initially misdiagnosed as a seizure disorder associated with syncope and treated with anti-epileptics for a neurogenic cause, before an ambulatory cardiac monitor revealed a sinister cardiogenic etiology.

**Case Presentation:**

An 87-year-old man with ischemic cardiomyopathy (LVEF 20%) and persistent atrial fibrillation presented for implantable cardioverter-defibrillator (ICD) evaluation following a ventricular fibrillation (VF) arrest. He had a history of recurrent syncope accompanied by muscle jerking and was initially treated with anti-epileptic drugs. However, further evaluation with mobile telemetry revealed ventricular arrhythmias, including nonsustained VT, VF, and asystole. Anti-epileptic medications were discontinued, and the patient was started on amiodarone. A cardiac resynchronization therapy defibrillator (CRT-D) was implanted, which successfully resolved his symptoms. Post-treatment, he remained asymptomatic, with no new VT/VF episodes detected at one week and three months during follow-up device checks.

**Conclusion:**

This case underscores the importance of considering cardiogenic causes in patients with syncope and seizure-like symptoms. Therefore, a multidisciplinary approach is essential for accurate diagnosis and management.

## 1 INTRODUCTION

Syncope and seizure disorders represent distinct clinical entities with overlapping presentations, often complicating the diagnostic process [1, 2]. Seizure disorders arise from abnormal cortical electrical activity, while syncope results from transient cerebral hypoperfusion, leading to a temporary loss of consciousness [3, 4]. Despite comprehensive clinical evaluations, differentiating these two conditions remains challenging, as both may present with convulsive activity and altered mental status. Approximately 3% of the U.S. population experiences recurrent convulsive episodes, with 20-30% attributed to underlying cardiogenic causes, including arrhythmias [5].

The episodic nature of symptoms associated with cardiogenic syncope further complicates diagnosis, as episodes are rarely captured on synchronized video telemetry with electroencephalography (EEG) and electrocardiography (EKG) [6, 7]. Failure to recognize cardiogenic causes promptly can result in significant morbidity, highlighting the importance of thorough cardiac evaluation in selected cases of suspected seizure disorders.

Tachy-bradyarrhythmias, a type of sick sinus syndrome arising from sinus node dysfunction and characterized by alternating episodes of tachycardia and bradycardia, are a significant but underrecognized cause of cardiogenic syncope [8]. These arrhythmias can induce transient global cerebral hypoperfusion, manifesting with features resembling epileptic seizures, such as myoclonic jerks, tonic spasms, and vocalizations. These presentations can lead to extensive neurogenic evaluations, misdiagnoses, and unwarranted treatments with anti-epileptic medications [9, 10].

In this report, we present the case of an elderly male with ischemic cardiomyopathy and persistent atrial fibrillation, whose recurrent syncopal episodes and altered sensorium were initially attributed to a seizure disorder. Subsequent investigations revealed an arrhythmogenic etiology. This case highlights the necessity of considering cardiogenic causes in patients presenting with syncope and seizure-like symptoms and underscores the importance of detailed monitoring and a multidisciplinary approach for accurate diagnosis and optimal management.

## 2 CASE PRESENTATION

### 2.1 History of Presenting Illness

**Fig. (1):**
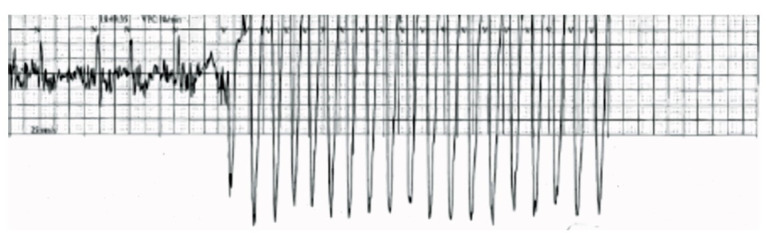
Telemetry strip from peripheral strip showing an episode of Ventricular Fibrillation (VF), followed by a period of asystole.

An 87-year-old man with a history of coronary artery bypass grafting (CABG), ischemic cardiomyopathy (LVEF 20%), and persistent atrial fibrillation (AF) presented to the hospital for implantable cardioverter-defibrillator (ICD) evaluation after sustaining a brief ventricular fibrillation (VF) arrest at another institution (Fig. **1**).

In the month leading up to this presentation, the patient experienced multiple syncopal episodes characterized by altered sensorium and muscle-jerking activity, leading to repeated hospitalizations at the outside hospital (OSH). The initial cardiac workup at the OSH was unrevealing with none of the following: documented ischemia on EKG, arrhythmias on short-term inpatient telemetry, regional wall motion abnormalities on echocardiogram, or reproducible symptoms *via* tilt-table testing. These episodes of altered sensorium, ranging from minutes to hours, were often followed by postictal-like states, and on one occasion, urinary incontinence was noted before eventually returning to baseline mental status. Based on these findings, a diagnosis of seizure disorder was made, and the patient was started on levetiracetam.

Given the patient’s significant cardiac history, he was discharged from OSH with a Mobile Cardiac Outpatient Telemetry (MCOT) system for further monitoring. Despite anti-epileptic treatment, he sustained a syncopal collapse 48 hours after discharge, accompanied by vomiting and concerns for aspiration, leading him to return to the OSH. MCOT data from this episode documented ventricular tachycardia (VT), self-terminating VF, and a period of asystole.

**Fig. (2):**
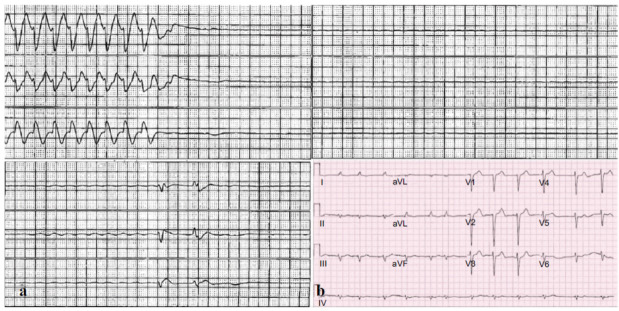
Telemetry strip from outside hospital (lead V1) showing initial rhythm being AF (irregularly irregular narrow QRS complex) followed by a premature ventricular complex (PVC) degenerating into ventricular fibrillation (VF) followed by a period of asystole. (**a**) Three lead cardiac monitor showing multiple episodes of monomorphic ventricular tachycardia followed by a prolonged period of ventricular pause (~ 12.5 second) ensuing into an extended period of slow junctional escape and occasional PVCs (Upper and lower left). (**b**) Baseline EKG showed rate controlled atrial fibrillation, LBBB with no ischemic ST-T wave changes.

After being admitted to the OSH for a workup of the syncopal episode, the patient had another episode of acute change in mentation with myoclonic jerks, which correlated with the episode of VF on the telemetry. Return of Spontaneous Circulation (ROSC) was achieved before ACLS could be started (Fig. **2**). At this point, the patient was transferred to our hospital.

Upon arrival, he was hemodynamically stable, not on vasopressors or inotropes, but somnolent while following commands. Cardiac auscultation revealed an irregular rhythm without murmurs, while lung examination demonstrated bilateral rales. Pitting edema was observed in both lower extremities up to the mid-tibia with a warm profile. Baseline 12-lead EKG revealed atrial fibrillation and left bundle branch block (LBBB) (Fig. **2**).

### 2.2 Past Medical History

The patient’s medical history included coronary artery disease (status post-CABG), ischemic cardiomyopathy, chronic systolic heart failure with a Left Ventricular Ejection Fraction (LVEF) of 20%, persistent AF, renal cell carcinoma, pulmonary embolism on chronic anticoagulation with warfarin, and hypertension. A primary prevention ICD had been previously recommended but was declined by the patient.

### 2.3 Investigations

Initial evaluations post-arrest and at our institution included serial EKGs, which confirmed AF with LBBB and no ST and T wave abnormalities suggestive of ischemia (Fig. **2**). Chest X-ray revealed bilateral infiltrates and a right-sided pleural effusion. Laboratory results showed elevated Pro-B-type natriuretic peptide (NT-BNP) at 3,328 pg/ml, serum creatinine at 1.22 mg/dl, and normal troponin T at 0.04 ng/ml without an upward trend. Initial lactic acid was elevated at 2.1 mmol/L but normalized within eight hours. Computed tomography of the head was negative for intracranial abnormalities. Transthoracic echocardiography demonstrated severely reduced LVEF (15-19%) with regional severe hypokinesis of the basal-mid inferior and inferolateral wall segments in addition to global hypokinesis and abnormal septal motion (Videos **S1** and **S2**). The right ventricle was dilated with normal systolic function. A left heart catheterization was performed additionally, which confirmed patent bypass grafts and stable coronary artery disease, unchanged from two years prior.

The MCOT data was retrieved on day two, which provided critical diagnostic insight, documenting recurrent episodes of nonsustained and sustained VT (~160 BPM) and self-terminating VF (Fig. **1**). Each episode of VT/VF led to a period of asystole (ranging from 4.4 to 12 seconds), followed by a longer period of complete heart block with junctional escape rhythm (~10-15 BPM lasting up to a minute). These arrhythmias were temporally associated with the patient’s symptoms, as confirmed by family history and careful correlation with clinical events.

### 2.4 Differential Diagnosis

The differential diagnosis for patients who present recurrent syncope and altered mental status is broad, including neurogenic causes, such as a transient ischemic attack, carotid artery stenosis, carotid sinus hypersensitivity, intracranial space-occupying lesions, migraine and sleep disorders, such as narcolepsy and benign sleep myoclonus. Other notable aetiologies include psychological causes (*e.g.*, psychogenic seizure, panic attack, epilepsy, conversion disorder), metabolic disturbances (*e.g.*, alcohol intoxication, hypoglycemia, hypoxia, psychoactive drugs), and cardiogenic causes (*e.g.*, vasovagal syncope, cardiac arrhythmia, valvular heart disease, orthostatic hypotension, and cardiomyopathies). The ethical approval for this study was waived by the International Review Board.

### 2.5 Management

After attributing the patient’s symptoms to a tachy-bradyarrhythmia etiology, anti-epileptic medications were discontinued on day two of admission. He was initiated on amiodarone for VT/VF suppression, and guideline-directed medical therapy (GDMT) for heart failure was subsequently optimized. Given his reduced LVEF of 15-19%, history of VF, and the need for ventricular pacing, the patient was deemed an appropriate candidate for a cardiac resynchronization therapy defibrillator (CRT-D), which was successfully implanted on hospital day three without complications.

### 2.6 Follow-up

After completing treatment for aspiration pneumonia, GDMT optimization, and CRT-D placement, the patient was discharged to home in good clinical condition on day 13. Post-discharge follow-up, including device checks at one week and three months, showed no new episodes of VT/VF, and the patient remained asymptomatic without recurrent seizure-like episodes.

## 3 DISCUSSION

Syncope remains a diagnostic challenge with overlapping clinical presentations, particularly when accompanied by seizure-like activity [5, 11]. Such presentations are easily misinterpreted as neurogenic seizures, often leading to unremarkable neurogenic investigations, such as EEG and brain imaging, as highlighted by Zaidi *et al.* [10]. While it has an annual incidence of approximately 6% and a lifetime prevalence of 42% [12], arrhythmogenic causes, such as tachy-bradyarrhythmias, are frequently underrecognized [13, 14].

The pathophysiology of tachy-bradyarrhythmias involves impaired sinoatrial node function, resulting in alternating periods of rapid and slow heart rates. These episodes lead to transient global hypoperfusion, precipitating symptoms, such as myoclonic jerks and altered mental status, challenging differentiation from epileptic seizures. Patel *et al.* highlighted that ictal bradycardia syndrome, or convulsive syncope, can mimic seizures, necessitating simultaneous EEG and cardiac telemetry to identify the underlying etiology.

Severe ischemic cardiomyopathy, the leading cause of HFrEF, predisposes patients to ventricular arrhythmias through myocardial fibrosis and electrical remodeling. Heart failure phenotypes, such as “warm and wet,” are associated with significant hypervolemia and increased mortality risk compared to “warm and dry” profiles (HR 1.83; *p* = 0.02) [15]. In this case, our patient presented with a “warm and wet” profile and altered mental status but improved following prompt stabilization. This underscores the importance of recognizing distinct hemodynamic profiles in the management of syncope.

Diagnostic clarity often hinges on advanced tools, such as MCOT and implantable loop recorders, which enable real-time correlation of arrhythmias with clinical symptoms [16, 17]. For instance, Elkattawy *et al.* [18] emphasized the pivotal role of telemetry in capturing arrhythmic events, such as bradycardia progressing to asystole or ventricular tachycardia during clinical episodes. These diagnostic modalities are essential given the episodic nature of arrhythmias and their tendency to elude routine evaluations, such as 12-lead ECG or short-term Holter monitoring.

The management of tachy-bradyarrhythmias includes addressing intrinsic causes, such as sinoatrial node fibrosis, and mitigating extrinsic triggers like metabolic disturbances or medications [19]. In patients with significant arrhythmias, interventions, such as dual-chamber pacemakers for sinoatrial node disease or cardiac resynchronization therapy defibrillators for life-threatening tachyarrhythmias, are indicated. These measures not only alleviate symptoms but also prevent life-threatening episodes of asystole or ventricular arrhythmias [20].

In this case, the patient’s symptoms were initially attributed to a neurogenic etiology, leading to unnecessary anti-epileptic therapy [5]. Only with MCOT findings of recurrent ventricular tachycardia, ventricular fibrillation, and prolonged sinus pauses was the correct diagnosis of tachy-bradyarrhythmias established, changing the management and outcome of our patient.

## CONCLUSION AND FUTURE PERSPECTIVES

Syncope, particularly in elderly patients with recurrent episodes and overlapping symptoms, such as altered mental status and seizure-like activity, demands a comprehensive diagnostic approach. This case highlights the importance of considering cardiogenic causes, such as tachy-bradyarrhythmia, in the differential diagnosis and utilizing advanced diagnostic tools like mobile cardiac telemetry and implantable loop recorders to capture elusive arrhythmic events. Early recognition and timely interventions, including cessation of inappropriate anti-epileptic therapy and appropriate device implantation, can significantly improve outcomes, underscoring the need for multidisciplinary collaboration and systematic evaluation in complex cases.

It is important to appreciate the differential diagnosis and conduct the initial evaluation for syncope. Recognizing the appropriate risk factors is crucial, and a cardiogenic cause for altered mental status should remain at the top of the differential diagnosis. Moreover, understanding the basic approach and selection criteria for implantable devices in the management of arrhythmogenic syncope is essential.

## Data Availability

Not applicable.

## LIST OF ABBREVIATIONS

AF: Atrial Fibrillation
CABG: Coronary Artery Bypass Grafting
CRT-D: Cardiac Resynchronization Therapy Defibrillator
GDMT: Guideline-Directed Medical Therapy
ICD: Implantable Cardiac Defibrillator
LBBB: Left Bundle Branch Block
LVEF: Left Ventricular Ejection Fraction
MCOT: Mobile Cardiac Outpatient Telemetry
OSH: Outside Hospital
ROSC: Return Of Spontaneous Circulation
VF: Ventricular Fibrillation

## STANDARDS OF REPORTING

CARE guidelines were followed.

## DECLARATION

During the preparation of this work, the authors used Grammarly to improve the language and grammar of the text. After using this tool/service, the authors reviewed and edited the content as needed and took full responsibility for the content of the publication.
